# Molecular Modeling Applied to the Discovery of New Lead Compounds for P2 Receptors Based on Natural Sources

**DOI:** 10.3389/fphar.2020.01221

**Published:** 2020-09-29

**Authors:** Anael Viana Pinto Alberto, Natiele Carla da Silva Ferreira, Rafael Ferreira Soares, Luiz Anastacio Alves

**Affiliations:** ^1^ Laboratory of Cellular Communication, Oswaldo Cruz Institute, Oswaldo Cruz Foundation, Rio de Janeiro, Brazil; ^2^ Laboratory of Functional Genomics and Bioinformatics, Oswaldo Cruz Institute, Oswaldo Cruz Foundation, Rio de Janeiro, Brazil

**Keywords:** natural products, P2 receptors, virtual screening, molecular dynamics, homology modeling, drug discovery, molecular modelling

## Abstract

P2 receptors are a family of transmembrane receptors activated by nucleotides and nucleosides. Two classes have been described in mammals, P2X and P2Y, which are implicated in various diseases. Currently, only P2Y12 has medicines approved for clinical use as antiplatelet agents and natural products have emerged as a source of new drugs with action on P2 receptors due to the diversity of chemical structures. In drug discovery, *in silico* virtual screening (VS) techniques have become popular because they have numerous advantages, which include the evaluation of thousands of molecules against a target, usually proteins, faster and cheaper than classical high throughput screening (HTS). The number of studies using VS techniques has been growing in recent years and has led to the discovery of new molecules of natural origin with action on different P2X and P2Y receptors. Using different algorithms it is possible to obtain information on absorption, distribution, metabolism, toxicity, as well as predictions on biological activity and the lead-likeness of the selected hits. Selected biomolecules may then be tested by molecular dynamics and, if necessary, rationally designed or modified to improve their interaction for the target. The algorithms of these *in silico* tools are being improved to permit the precision development of new drugs and, in the future, this process will take the front of drug development against some central nervous system (CNS) disorders. Therefore, this review discusses the methodologies of *in silico* tools concerning P2 receptors, as well as future perspectives and discoveries, such as the employment of artificial intelligence in drug discovery.

## Introduction

Plants have been used as medicine for over 60,000 years and form the basis of traditional medicines worldwide, including Chinese Medicine, Korean Medicine, Kampo (Japan), Ayurveda and Unani (India) (Yuan et al., 2016). Currently, about 20,000 medicinal plants are used in 91 countries worldwide, including Brazil, China, France, Germany, and the United Kingdom (Sasidharan et al., 2011).

Natural products have been explored in drug development since the beginning of the 19th century. The first isolated compound from natural products was morphine, isolated from the opium plant by Friedrich Sertürner in 1805 and commercialized by Merck in 1826 (Ji et al., 2009; Yuan et al., 2016). Currently, several synthetic compounds whose original structures are based on natural products are used in the treatment of numerous diseases, including hypercholesterolemia (e.g. simvastatin and lovastatin), hypertension (e.g. captopril and enalapril), cancer (e.g. taxol and docetaxel), and infection (e.g. penicillin and amphotericin B) (Calixto, 2019). Furthermore, approximately 35% of global medicines directly or indirectly originate from natural products, including plants, animals, and microorganisms. In the field of cancer and infectious diseases, up to 60 to 75% of drugs originate from natural products, respectively (Gullo et al., 2006; Calixto, 2019).

Newman and Cragg (2016) conducted a search of the FDA database to investigate the amount of new chemical entities (NCEs) based on natural products that emerged between 1981 and 2014. Among 1,562 NCEs, 16% have a biological origin, 4% were unaltered natural products, 1% comprised botanical drugs, 21% suffered semisynthetic modification, and 4% were synthetic drugs with a pharmacophore similar to that of a natural product. These drugs display wide applications in therapy, including in the treatment of neurodegenerative, cardiac, metabolic, infectious, and inflammatory diseases (Newman and Cragg, 2016). In addition, in 2007 at least 91 plant-derived molecules were used in clinical trials worldwide for the treatment of several diseases (Saklani and Kutty, 2008).

The use of natural products in the process of drug discovery has immeasurable value. First, natural products display a great diversity of chemical structures, acquired over thousands of years as a result of a co-evolution within communities. Second, many of these structures have not yet been reported and may constitute a model for the synthesis of novel drugs, which could be modified by chemists to improve characteristics including efficacy, solubility, and stability in the human body (Ji et al., 2009; Calixto, 2019). These modifications and many other features are included in a field of cheminformatics, discussed later in this paper.

The process of drug discovery using natural products exhibits some obstacles. One is the need to perform various processes until the determination of the active molecule since test samples often consist of extracts or fractions (Siddiqui et al., 2014; Chen et al., 2017). Several studies have ended before they were able to conduct active molecule purification, possibly due to the high complexity of such mixtures. The therapeutic activity found in extracts may be in some cases due to the synergistic and simultaneous action of several molecules (Shen, 2015; Thomford et al., 2018). Isolated molecules are often not available in sufficient quantities for use during high throughput screening campaigns (Siddiqui et al., 2014). Lack of selectivity might also limit research, because the different molecules that are present in extracts can bind to several cellular targets (Shen, 2015). Finally, legal regulations may impact natural product research, i.e. the certification required to use biodiversity information in research (Siddiqui et al., 2014).

The evolution of bioinformatics and cheminformatics in conjunction with analytical technologies has revolutionized the field of natural product research by enabling the rapid detection of hits through virtual screening (VS) and facilitating the isolation and structural elucidation of active molecules (Shen, 2015). Virtual databases can be tested using the molecular docking technique, allowing for the selective analysis of test molecule and target pharmacological interactions, saving time and the expense of reagents and lab consumables (Fischer et al., 2014). Several virtual libraries in which thousands of molecules of synthetic and natural origin are already registered and available. Some databases even offer plant-derived molecules that are used in Traditional Chinese or African Medicine, such as the Traditional Chinese Medicine Integrated Database (TCMID) and the African Medicinal Plants Database (AfroDb) (Chen et al., 2017). These techniques and approaches to virtual databases will be discussed in further detail in this study.

## Purinergic Receptors as Targets for Drug Development

Extracellular nucleotides activate plasma membrane receptors in mammalian species, termed P2 purinergic receptors. P2 receptors are categorized into two classes: P2Y, which comprises G protein-coupled receptors; and P2X, which consist of ionotropic receptors (Burnstock and Kennedy, 1985). The P2Y class contains eight members described in humans: P2Y1, P2Y2, P2Y4, P2Y6, P2Y11, P2Y12, P2Y13, and P2Y14 (Abbracchio et al., 2006). P2X includes seven members (P2X1-P2X7) (Fountain, 2013). As ionotropic receptors, P2X members open an ion channel permissive to cations when activated. The P2X5 receptor is the only exception since it is more permeable to anions than cations. The P2X7 receptor possesses the unique characteristic of membrane pore formation, which is activated at high ATP concentrations (above 100 μM). This pore can transport molecules of up to 900 Da to the intra or extracellular medium, according to an electrochemical gradient. These molecules include the fluorodyes propidium iodide, lucifer yellow, ethidium bromide, and YO-PRO-1 (Coutinho-Silva et al., 1996; Coutinho-Silva and Persechini, 1997; Persechini et al., 1998; Alves et al., 2014; Pacheco et al., 2016).

P2 receptors are broadly expressed in humans, including in the immune, respiratory, cardiovascular, and central nervous systems, as well as gastrointestinal and urinary tracts (Burnstock and Knight, 2004). The P2 receptors in these tissues have important physiological functions. In airways, for example, P2 receptors promote surface lubrification, mucus hydration and secretion, and ciliary beat (Jacobson and Boeynaems, 2010). The ATP released to the extracellular medium alerts the immune system to danger and generates a chemotaxis gradient for immune cells to the injury site. Activation of these receptors results in cytokine release, reactive oxygen species (ROS) formation, phagocytosis, and antigen presentation, which may contribute to chronic inflammation (Rayah et al., 2012). The upregulation of P2 receptors in neurons and glial cells has been associated with pain development (da Silva Ferreira et al., 2019). The P2 receptors expressed on platelets are also associated with platelet aggregation and are important targets for anti-thrombotic drugs in recent decades (Zhang et al., 2017; da Silva Ferreira et al., 2019).

In terms of structure, P2Y presents seven transmembrane domains, a carboxyl terminus into the intracellular milieu and an amino terminus facing the extracellular compartment (Zhang et al., 2014a; Zhang et al., 2014b; Zhang et al., 2015). P2X receptors are structurally much more simple, presenting a monomer with two transmembrane domains, an extracellular loop, and both amino and carboxy termini facing the intracellular compartment (Hattori and Gouaux, 2012; Habermacher et al., 2016; Kasuya et al., 2017). The functional protein works as a trimer that can be formed by equal subtypes (homotrimers) or different subtypes (heterotrimers). Only a few functional heterotrimers have been described in the literature, namely P2X1/2, P2X1/5, P2X2/3, P2X2/5, P2X2/6, P2X4/6, and P2X4/7 (Slater et al., 2000; Saul et al., 2013). It is important to note that P2X6 is the only subtype that does not form functional homotrimers (Collo et al., 1996; King et al., 2000; Jones et al., 2004).

The studies discussed above were reconfirmed in recent years by the crystallization of some P2 receptors. The first one resolved by x-ray crystallography was the zebrafish P2X4 (zfP2X4) in 2009 (Kawate et al., 2009). Next, other P2 receptors were resolved both by x-ray crystallography and cryo-electron microscopy, including the human P2Y12 (hP2Y12) (Kiselev et al., 2014), human P2Y1 (hP2Y1), human P2X3 (hP2X3), chicken P2X7 (ckP2X7), giant panda P2X7 (gpP2X7), and rat P2X7 (rP2X7) (Hattori and Gouaux, 2012; Zhang et al., 2014a; Zhang et al., 2014b; Zhang et al., 2015; Habermacher et al., 2016; Karasawa and Kawate, 2016; Mansoor et al., 2016; Kasuya et al., 2017; McCarthy et al., 2019).

**Figure 1:**
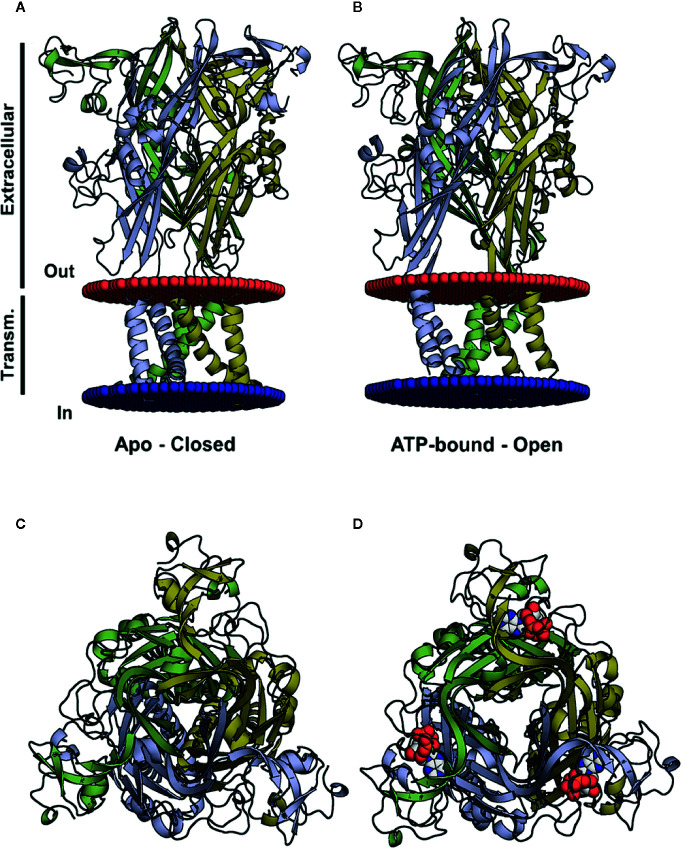
Three-dimensional structures of the closed and open (ATP-bound) P2X4 states. **(A**, **B)** Crystal structure of the zfP2X4 (PDB code 4DW0) in different views, apo and open, with the transmembrane helices colored according to the subunit colors in blue, yellow, and green. ATP molecules are displayed as a van der Waals representation. **(C)** The same structure as in **(A)**, viewed from the top along the axis of the central channel. **(D)** The same structure as in **(B)**, seen from the top along the axis of the central channel, with ATP bound to the pharmacophore site.

In recent decades there have been important breakthroughs in research on purinergic receptors, resolved by the elucidation of the structure of zfP2X4 (**Figure 1**) (Kawate et al., 2009). This allowed for the clarification of the agonist pocket, the protein organization in the trimeric assembly, and the folding of its subunits since crystallization was performed in both open and closed states (Kawate et al., 2009; Kawate et al., 2011). Moreover, it was possible to postulate that the passage of ions could occur through an adjacent region close to the membrane (**Figure 1**), i.e., fenestrations, and not by the central receptor pathway (Habermacher et al., 2016). The zfP2X4 structure was compared to a dolphin and some papers mention the left flipper, right flipper, tail, body, and head when indicating the studied portion of the protein (Kawate et al., 2009; Kawate et al., 2011). These findings enabled research into new drugs to treat P2X-related diseases such as chronic inflammation and pain (Cockayne et al., 2005; Honore et al., 2006; Donnelly-Roberts and Jarvis, 2007).

**Figure 2:**
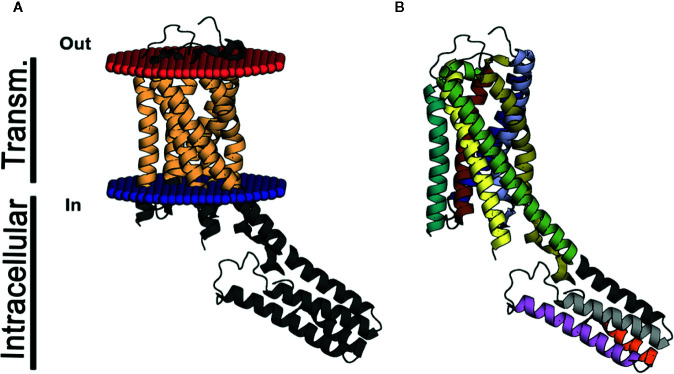
3D structure of P2Y12. The structure in **(A**, **B)** represents the P2Y12 (PDB code 4PXZ) crystal in the lateral view. **(A)** indicates the helix in light brown represents the transmembrane domain of P2Y12 and **(B)** exhibits the same segments represented in dark blue, light blue, red, yellow, brown, light brown, and green. The intracellular helices are represented in **(A)** in dark gray and in **(B)**, in gray, light gray, orange and purple. The part of the transmembrane domain that extends to the intracellular space is displayed in yellow and green.

The resolution of the P2Y12 receptor by x-ray crystallography facilitated new insights into its structure, including the presence of two binding pockets, one for nucleotide ligands and another for non-nucleotide ligands (**Figure 2**) (Zhang et al., 2014a; Zhang et al., 2014b). Although the binding mode is different, other studies have had similar findings for the P2Y1 receptor (Zhang et al., 2015). Currently, four P2Y12 drugs are being used in clinical therapy for thrombosis prevention: clopidogrel (Plavix^®^), prasugrel (Effient^®^), ticagrelor (Brilinta^®^), and cangrelor (Kengreal^®^) (Savi et al., 2006; Cattaneo, 2007; Deflorian and Jacobson, 2011; Jacobson et al., 2011; Paoletta et al., 2015). These data are crucial in the context of antithrombotic drugs facilitating the exploration of new targeted therapies based on the ligand pocket.

Recent advances in research on the P2 receptor structure are particularly significant for bioinformatics, a field of science that is growing exponentially (Hillisch et al., 2015; Ferreira and Andricopulo, 2018), and 3D resolved structures, alongside advances in the development of algorithms have allowed for more accurate predictions.

## 3D Structures and Molecular Modeling Techniques Applied to Drug Discovery

In recent years, computer programs and algorithms have become more efficient at processing complex data. Artificial intelligence (AI) regularly outperforms humans, for example, an AI recently beat the best player at Go, a Chinese game considered more difficult than chess (Silver et al., 2018). Today, many algorithms function in the Windows operating system, although it is more common to operate in linux kernel base of several open source system, an open-source operating system.

**Figure 3:**
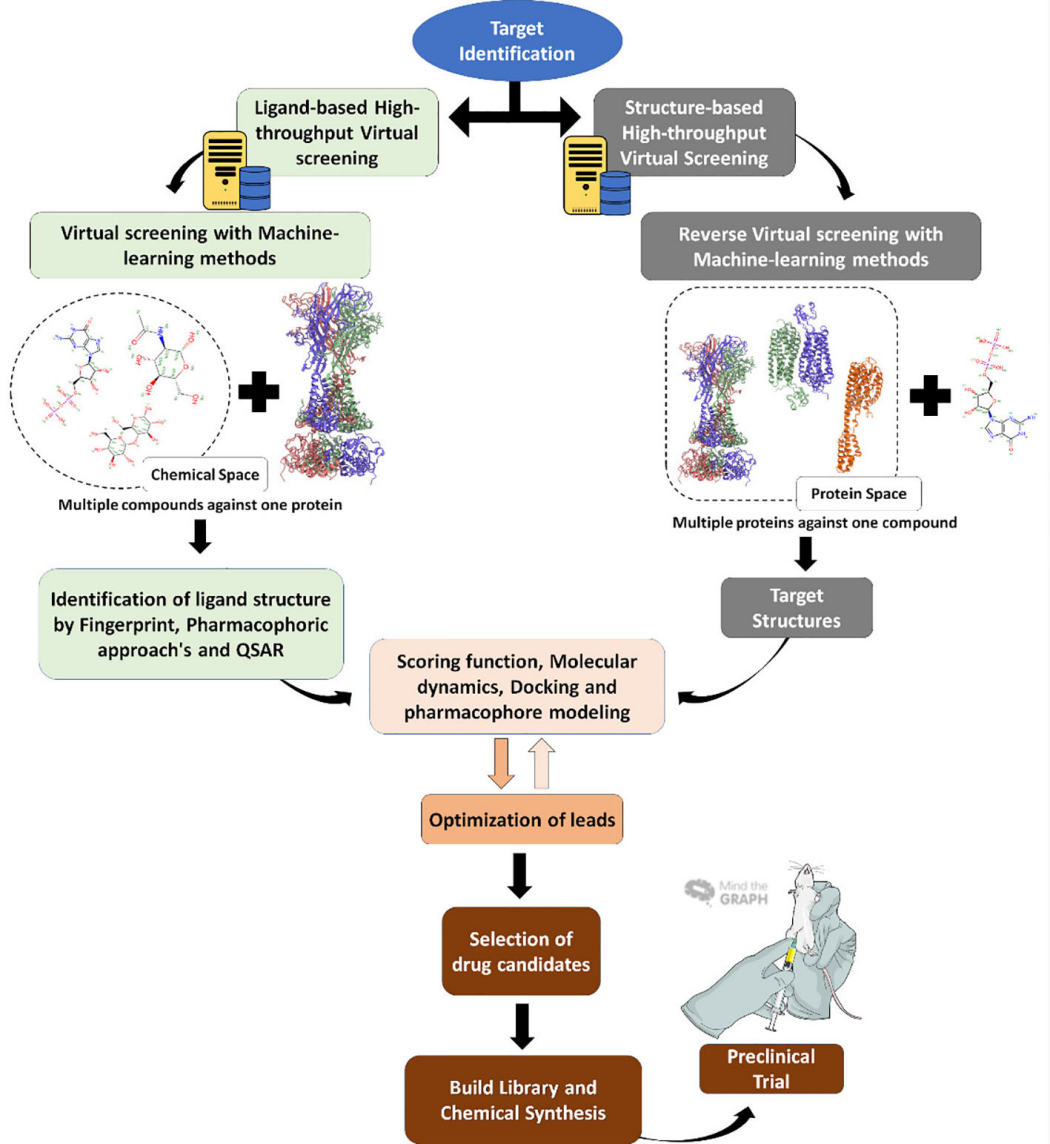
Virtual screening workflow. Virtual screening steps since target identification, usually a protein (right) or the reverse, with the target being the molecule, until the final step of *in vitro* and *in vivo* tests.

Programs are often used to simulate the steps in classical approaches to high throughput screening (HTS) assays, by downloading molecule databases such as plant metabolites and secondary natural products and adding them to a list that enables them to conduct a virtual screening. This is followed by assessments of toxicity, absorption, solubility, lead-likeness, and other clinical parameters, displayed in **Figure 3**. The entire flow is low cost and faster than the HTS used by the industry to discover new drugs and classic HTS is starting to be exchanged for VS in the search for new drugs (Ekins et al., 2007; Biggin and Bond, 2008; Morris and Lim-Wilby, 2008). In the future, molecular modeling will take the place of HTS in research groups and the pharmaceutical industry. The next sections detail each form of molecules and other ligand investigations concerning specific proteins.

## Comparative Protein Structure Modeling

In protein structure determination, the cloning, expression, and purification steps often exhibit problems that slow progress. Similarly, crystallization methods also display methodological and technical difficulties that can delay or hinder the obtaining of a crystal. In this context, predictive methods such as homology modeling, also known as comparative modeling, save time and reduce costs.

Comparative modeling is a technique that generates a 3D model of a protein from an amino acid sequence (target sequence) using one or more related, known structures (templates). Since this method is based on similarities in the amino acid sequence from two proteins that belong to the same family, both are expected to show some degree of similarity in 3D structure (Mosimann et al., 1995). The protein structure of the same family is more highly conserved than their amino acid sequences (Forrest et al., 2006).

The accuracy, applicability, and success of comparative modeling depends on structural divergence during the evolutionary time between template and target and also on the extent of sequence similarity. Usually, the sequence identity requires 70% or higher similarity, for it to be considered a reliable prediction. Inaccurate models generally display sequence identities lower than 30% (Forrest et al., 2006).

The prediction of membrane proteins has fewer restrictions in terms of sequence identity, i.e., approximately 30% or higher similarity. This also occurs even if the extracellular domain prediction has low accuracy (Forrest et al., 2006). The inaccuracy of transmembrane domains from the model can be related to problems inherent to the technique and structural deficiencies such as the presence of detergents to solubilize the template structure.

Despite its limitations, the comparative modeling approach comprises some solutions to minimize occurrences that lead to inaccuracies. Many user-friendly servers with automated web interfaces currently provide comparative modeling for non-specialist users, meaning that results can be analyzed with no software installation. Common comparative modeling programs include SWISS-MODEL (pioneered automated server) and ROSETTA (Simons et al., 1999; Webb and Sali, 2016; Waterhouse et al., 2018).

Comparative modeling consists of four steps: a) a comparison between the sequences of the known structure and the homologous sequence to maximize template reliability; b) the alignment of the target sequence with one or more selected templates; c) building 3D models based on these alignments; and d) quality evaluation of structure models to perform physicochemical refinements (Kryshtafovych et al., 2005; Waterhouse et al., 2018).

Notwithstanding the difficulties encountered in transmembrane protein studies, several studies have presented models of predicted structures for P2X receptors. This has aided understanding of critical amino acid residues and important domains not completely verified by experimental assessments. In this sense, P2X receptors can be studied from a mutation standpoint to discover critical amino acid residues. These mutations allow studies on the mobility of ion channels (i.e., opening and closing) as well as analyses regarding ATP and protein interactions (Yan et al., 2006). For example, the substitution of glycine for alanine in the lower body of the P2X4 receptor resulted in a more rigid structure, decreased ATP sensitivity, slower activation, and desensitization (Habermacher et al., 2016). The comparative protein structure modeling method is useful in predicting the 3D structures of P2 receptors that do not have crystallographic data yet. These 3D structures are used in molecular docking assays to discover new ligands for the receptors, and this strategy has been adopted by several research groups. In order to understand the interactions between P2X receptors and drug-like compounds, Dal Ben et al. (2015) studied the interactions of this complex using comparative model structures of human and rat P2X receptors based on a zfP2X4 crystallography structure template. Molecular docking of ATP and P2X agonists were performed in the ATP-binding site (Dal Ben et al., 2015). Chen et al. (2011) constructed a human P2Y12 model based on the β1 adrenergic receptor from *Meleagris gallopavo*. Using this structure, they performed a virtual screening campaign from the ZINC database and found nine potential P2Y12 receptor antagonists (Chen et al., 2011). Rafehi et al. (2017a) developed a P2Y4 structure based on the P2Y1 receptor and selected some anthraquinone derivatives compounds to perform molecular docking. The authors demonstrated that compound 61 (sodium 1-amino-4-[4-(2,4-dimethylphenylthio)-phenylamino]-9,10-dioxo-9,10-dihydroanthracene-2-sulfonate) presented the lowest IC_50_ for P2Y4, therefore constituting a potential antagonist for this receptor (Rafehi et al., 2017a). **Table 1** summarizes studies that apply the homology strategy.

**Table 1:** Comparative protein structure modeling methods in the structure prediction of P2 receptors.

P2 receptors	Homologous protein	Reference
Human P2Y1 and P2Y12	Bovine rhodopsin	(Costanzi et al., 2004)
Human P2Y1	Bovine rhodopsin	(Major and Fischer, 2004)
Human P2Y11	Bovine rhodopsin and human P2Y1	(Zylberg et al., 2007)
Human P2Y2	Bovine rhodopsin	(Hillmann et al., 2009)
Human P2Y12	β1 adrenergic receptor from *Meleagris gallopavo*	(Chen et al., 2011)
Rat P2X2 and human P2X5	Zebrafish P2X4	(Kawate et al., 2011)
Human P2Y12	Bovine rhodopsin, human A2A adenosine receptor and human C-X-C chemokine receptor type 4	(Deflorian and Jacobson, 2011)
Rat P2X2	Zebrafish P2X4	(Hausmann et al., 2013)
Human P2Y14	Human P2Y12	(Kiselev et al., 2014)
Human and rat P2X1, P2X2, P2X3, P2X4, P2X5 and P2X7	Zebrafish P2X4	(Dal Ben et al., 2015)
Human P2Y14	Human P2Y12	(Trujillo et al., 2015)
Human P2Y14	Human P2Y12	(Kiselev et al., 2015)
Human P2Y14	Human P2Y12	(Junker et al., 2016)
Human and rat P2X7	Zebrafish P2X4	(Caseley et al., 2016)
Human P2X7	Zebrafish P2X4	(Pippel et al., 2017)
Human P2Y2	Human P2Y1	(Rafehi et al., 2017a)
Human P2Y2	Human P2Y1	(Rafehi et al., 2017b)
Mouse P2X7	Giant panda P2X7	(Pasqualetto et al., 2018)
Rat P2X4	Zebrafish P2X4	(Pasqualetto et al., 2018)
Human P2Y6	Human P2Y1 and P2Y12	(Jacob et al., 2018)
Human P2X4	Zebrafish P2X4	(Dhuna et al., 2019)
Human P2X7	Zebrafish P2X4	(Bidula et al., 2019)
Human P2Y14	Human P2Y12	(Wang et al., 2020)
Human, mouse, rat and zebrafish P2X4	Zebrafish P2X4	(Reyes-Espinosa et al., 2020)

## Molecular Docking and Virtual Screening

The binding molecule (ligand) has its rotational or translational space fathomed while the receptor remains rigid, usually to save computational time. This fact has guided several studies, enabling them to produce protein structures through experiments that apply crystallography or comparative modeling. The basis of the molecular docking method is the use of a search algorithm and score function that generates the ligand pose. Currently, it is possible to use various programs with different algorithms to compare the ligands’ pose. These programs include AutoDock (Morris et al., 2009), DOCK (Allen et al., 2015), Glide (Friesner et al., 2004), and GOLD (Jones et al., 1997). A comparison of results from different software programs may provide new questions and information concerning the assessed molecule.

Aiming to minimally converge the energy of the ligand, the algorithm evaluates its conformation recursively. A scoring function is applied to estimate the energy related to a specific conformation for a posterior rank (Yan et al., 2006). Docking programs generally sum the electrostatic potential and van der Walls energies to rank conformations.

Molecular docking has proven particularly important to research on the interaction between different molecules and pharmacological targets such as receptors. However, this methodology can be applied to screen large chemical libraries concerning a specific therapeutic target to find new drugs. This broad search, using billions of compounds by the computational approach, is termed virtual screening or *in silico* screening (Spyrakis and Cavasotto, 2015). A structure-based virtual screening can be performed using the molecular docking method, allowing for the evaluation of millions of similar compounds. Despite this, only a small fraction of compounds from the top-ranking conformations can be examined for interaction patterns and prioritized for purchase or synthesis (Spyrakis and Cavasotto, 2015).

One of the benefits of using this approach is the low computational power needed to perform a run and fast data acquisition, i.e., some conformations can be detected and ranked in a few minutes (Chen, 2015). However, the analyzed receptor is inflexible, which can produce inaccurate data, and may not indicate the evaluated molecule as a drug and may instead be a candidate that requires re-evaluation through other experimental methodologies (Chen, 2015).

Using the 3D structures of P2X receptors, the molecular docking approach has been applied to search for the best drug candidates for clinical trials, which could be applied in the treatment of several diseases, including cancer, rheumatoid arthritis and endocrine conditions (Dal Ben et al., 2015). ATP and other ligands are described in research involving P2X receptors, implementing protocols that include ATP stabilization and reduction of its degradation by ectonucleotidases (Adelman, 1976; Evans et al., 1995). Nucleotide-derived molecules, suramin-like analogs, and irreversible antagonists have been used in molecular docking approaches, aiding in the prediction of a druggable ligand in drug research (Dal Ben et al., 2015).

Research on P2 receptors through virtual screening has provided interesting information about the structure and molecular interactions of these receptors. The molecular docking approach is also applied in the testing of ligands from P2 receptors in order to evaluate selectivity and affinity, revealing novel potential drugs. Recently, molecular modeling and mutagenesis have advanced the search for novel P2Y ligands (Jacobson et al., 2012). Costanzi et al. (2012) selected 110 hits among 250,000 compounds tested for the P2Y1 receptor. As they describe, these molecules appear to be present an antagonist behavior even in a low molar range but require optimization to improve physicochemical characteristics (Costanzi et al., 2012). Nofianti and Ekowati (2019) performed a screening campaign of 22 o-hydroxycinnamic derivatives aiming to discover novel antiplatelet candidates. These compounds demonstrated the ability to inhibit both P2Y12 and COX-1 receptors and presented pharmacokinetic characteristics that allow oral administration (Nofianti and Ekowati, 2019). Recently, Wang et al. (2020) performed a virtual screening, which intended to discover novel P2Y14 antagonists. They selected a total of 19 compounds with different structures to conduct *in vitro* tests and found that 10 molecules presented an IC_50_ lower than 50 nM. They even found that compound 8 inhibited caspase-1 activation and IL-1β release (Wang et al., 2020).

Reyes-Espinosa et al. (2020) conducted a screening campaign to evaluate the potential positive allosteric modulator (PAM) activity on P2X4 of 1,657 drugs approved by the Food and Drug Administration (FDA). They evaluated the activity of these drugs in four different species (human, mouse, rat, and zebrafish) and identified nine molecules with PAM activity and eight as potential negative allosteric modulators (NAM) (Reyes-Espinosa et al., 2020). Caseley et al. (2016) have also described three hP2X7 antagonists with micromolar potency (IC_50_ < 6 μM) in a screening of over 100,000 compounds concerning the hP2X7 ATP-binding site. These compounds significantly inhibited calcium mobilization, dye uptake, and cell death induced by P2X7 activation, demonstrating that computational analyses can corroborate experimental data (Caseley et al., 2016).

The molecular docking technique has also been used to assess the antagonistic or modulating activity of molecules from natural products, although relatively few studies have to date been carried out. Yi et al. (2017) performed an *in silico* docking analysis from compounds deposited in the Traditional Chinese Medicine Systems Pharmacology Database and Analysis Platform (TCMSP), which contains structure information from herbs and natural ingredients used to discover novel antithrombotic drugs from medicinal plants. After the exclusion of compounds that were not in accordance with Lipinski’s rule of five, the authors evaluated 1,656 compounds from 443 herbs. They focused on compounds from three herbs: cimicifugae (*Cimicifuga foetida* L.), ganoderma (*Ganoderma lucidum* Karst), and licorice (*Glycyrrhiza uralensis* Fisch), as some studies have suggested that they demonstrate anti-thrombosis activity (Yi et al., 2017). Liu et al. (2018) performed a similar screening campaign to find novel ligands for P2Y1 and P2Y12 receptors that could be used as antithrombotic drugs. They evaluated 253 compounds from Traditional Chinese Medicines and tested 11 hits through *in vitro* assays, including salvianolic acids from *Salvia militorrhiza* (Liu et al., 2018). **Table 2** demonstrates some studies that applied virtual screening, using the molecular docking strategy to discover novel ligands for P2 receptors.

**Table 2:** Virtual screening campaigns targeting P2 receptors by applying the molecular docking strategy.

P2 receptor	Software	Sample	Best hits	References
Human P2Y12	DOCK 6.0	ZINC database	Compounds 1(a-c)^1^, 2(a-c)^2^ and 3(a-c)^3^	(Chen et al., 2011)
Human P2Y1	MOE	Compounds from Life Chemicals catalog	Compound 2a	(Costanzi et al., 2012)
Human and rat P2X7	eHiTS version 12	ZINC12 database	Compounds C23, C40 and C60	(Caseley et al., 2016)
Human P2Y1	Discovery Studio Client V4.5	Herbs and compounds from TCM	Compounds from herbs *G. uralensis* Fisch, *C. foetida* L., and *G. lucidum* Karst	(Yi et al., 2017)
Human P2Y4	Glide	Anthraquinone derivatives	Compounds 61 (PSB-16133) and 64 (PSB-1699)	(Rafehi et al., 2017a)
Human P2Y1 and P2Y12	Glide XP	TCM compounds with antiplatelet aggregation activity	Salvianolic acids A, B and C from *Salvia militorrhiza*	(Liu et al., 2018)
Human P2Y12	Molegro Virtual Docker	o-hydroxycinnamic acid derivatives	o-hydroxycinnamic acid derivatives (OCA1a–22a)	(Nofianti and Ekowati, 2019)
Human P2Y14	Glide	ChemDiv database	Compound 8	(Wang et al., 2020)
Human, mouse, rat and zebrafish P2X4	Autodock 4.2 and Autodock Vina	FDA-approved drugs deposited on ZINC15 database	Compounds A(1-13)^4^, B(1-8)^5^, and C(1-9)^6^	(Reyes-Espinosa et al., 2020)

^1^Classified as lead-like compounds, ^2^classified as fragment-like compounds, ^3^classified as drug-like, ^4^classified as allosteric modulators, ^5^classified as negative allosteric modulators, and ^6^classified as positive allosteric modulators.

Molecular docking has also been applied to evaluate the effect of two diterpenoids (tanshinone II-A and cryptotanshinone) from *Salvia milthiorriza* Bunge on human P2Y12. The analyses revealed that they interact with the binding site of this receptor and can inhibit *in vitro* platelet aggregation (Maione et al., 2015). Dhuna et al. (2019) have examined the activity of ginsenosides from *Panax ginseng*, a traditional Chinese medicinal plant, as positive allosteric modulators of the P2X4 receptor. These compounds enhanced Ca^2+^ influx and ATP-induced currents in HEK-hP2X4 cells, and docking data indicates that they bind to the central vestibule region of P2X4 (Dhuna et al., 2019). Bidula et al. (2019) also evaluate the activity of these ginsenosides on P2X7 using the molecular docking strategy, since previous studies demonstrated that these compounds act as positive allosteric modulators for this receptor. Docking data has demonstrated that the ginsenoside binding site is located within the central vestibule of P2X7 and some mutations in the amino acids from this region have resulted in the loss of dye uptake potentiation, calcium mobilization, ATP-induced current responses, and cell death (Bidula et al., 2019).

## Molecular Dynamics

Structure-based methods rely on a single pose of the target protein. The utilization of a single structure of a target protein is a major limitation for a detailed analysis of a given protein (Ivetac and Andrew McCammon, 2011). However, with advances in computational power, structural flexibility can be added to several methods that were impossible before. The classical molecular dynamics simulation is one of the most applied approaches to analyzing protein and ligand motion in the complex.

Molecular dynamics (MD) use Newton’s motion equation to progressively determine the energy states and conformational in the function of a feasible time scale (picoseconds, nanoseconds, or microseconds) (Spyrakis and Cavasotto, 2015). By obtaining information at the molecular level, the addition of temperature and pressure parameters to classical MD has provided new ways of carrying out studies and interpreting experiments (Rapaport and Rapaport, 2004; Durrant and McCammon, 2011).

MD is significantly cheaper in comparison to current computational methods and techniques, which tend to involve more detail, for example, quantum mechanics, molecular mechanics (QM/MM), or quantum chemistry (MD/QC). One explanation for this is that the Schrödinger equation is used in quantum methods. This equation represents the electron-nuclear in relation to static nuclei, while the classical MD uses an average field surrounding the atom nuclei to describe the electrons (Armunanto et al., 2003; Walker et al., 2008; Brunk and Rothlisberger, 2015).

Although computational power has greatly increased over the years, time scales were still a limitation. Some biological events require hundreds of microseconds to manifest, making classical MD unable to follow the event (depending on the system), particularly when simulations occur in a complex environment such as ion transport through a transmembrane protein. This system comprises a significant number of atoms (over 200,000) including the receptor, lipids in a bilayer configuration, neutralizing ions, a possible ligand, and mostly water molecules. In addition, the conformation of the ion channels tends to the closed state, due to their lower energy configuration (Bernardi et al., 2015).

Concerning these limitations of time, some studies have demonstrated interesting structural information, for example, interactions in the ATP binding site of the zfP2X4, which can determine some hydrophobic interactions between the left flipper and the dorsal fin, producing a downward movement of the left flipper and upward motion of the dorsal fin (Zhao et al., 2014). Lateral fenestrations have also been described as a gateway to ion passage through the channel through MD, later confirmed in cysteine accessibility assay experiments (Kawate et al., 2009; Hattori and Gouaux, 2012).

Due to the limitations of time, relatively few studies have applied or implemented classical MD. Coarse-grained simulation methods and enhanced sampling methods, such as metadynamics, simulated annealing, and replica-exchange molecular dynamics, are also available. Metadynamics can solve time scale problems depending on the analysis proposal. These methods are an alternative for simulating ion channels and study movements that occur in less than microseconds. The cheaper computational costs of coarse-grained methods are a consequence of the reduction of the number of degrees of freedom of the system, as some interactions can be removed to eliminate resources that are otherwise used to represent all atoms of the system. Additionally, an enhanced sampling method can be implemented to separate high and low-energy conformations to cross the high-energy barriers imposed in some biological systems. As an example of this technique, a coarse-grained simulation of an rP2X2 within a lipid bilayer is indicated by the interposition between lipids and alpha helices of the transmembrane region, which is representative of the stabilization function of these molecules in maintaining the open state of the receptor (Grimes and Young, 2015; Caseley et al., 2016).

In the field of drug discovery and development, MD has been used extensively for the refinement and optimization of constructed P2 receptor homology models to build templates for molecular docking assays (Zylberg et al., 2007; Trujillo et al., 2015; Junker et al., 2016; Liu et al., 2018). MD simulations have also been used in the evaluation of structure-function relationships between the binding pocket of the P2 receptor of interest and the candidate hits obtained through docking assays (Zhou et al., 2017).

## Artificial Intelligence

Cheminformatics is an area that applies several different computational methodologies to solve problems related to chemical information (Gasteiger, 2016). One of these methods, Artificial intelligence (AI), is believed by several researchers to be a breakthrough, representing a Fourth Industrial Revolution (Xu et al., 2018). The definition of AI is an area of intense debate, as also observed regarding the definition of human intelligence (Dobrev, 2005; Kok et al., 2009). Nevertheless, AI exhibits some striking features that are, in general, attributed to human intelligence, as measured by the well-known Turing test (Kok et al., 2009). These include automated reasoning, knowledge representation, natural language processing, and machine learning (ML). As in other areas, AI is applied with ML algorithms, an operational branch of AI.

The great advantage of ML algorithms is the capacity to rapidly make a decision based on a dataset with real examples. This is due to a large increase in computation processing in the last years, with the new graphics processing unit (GPU) heightening the capacity of parallel processing, and tensor processing unit (TPU), made to function with ML algorithms. These allow for the identification of several new molecules exhibiting activity in human systems, and thus decreasing the cost of new drugs placed on the market (Lavecchia, 2015; Esteva et al., 2019; Rifaioglu et al., 2019). Diverse ML algorithms have been used to discover new drugs (Carpenter et al., 2018), including Support Vector Machines (SVM), Random forest, k-nearest neighbors, Naïve Bayesian, decision trees, and deep neural networks. Most studies have applied SVM and deep neural networks.

SVM was first established to study chemical compounds in 2001 by two different groups, namely Burdidge and collaborators and Czerminski and collaborators, based on theories by Cortes and Vapnik (1995). The principles of SVM and its applications are explained by Maltarollo et al. (2019). It is important to note that SVM can be used to predict interactions between ligand and receptors, using physicochemical features, protein and compound descriptors, irrespective of structural information.

Deep neural networks are a subtype of artificial neural networks inspired by how neurons communicate with each other. Despite the complexity of the human brain, this algorithm is the only one that learns, using backpropagation to detects results “equal” or similar to the training dataset. This type of network is constructed with several neurons in hidden layers, and the weight of the networks can simulate inhibitory and excitatory synapses, thus leading to algorithm “plasticity” (Carpenter et al., 2018).

ML algorithms in drug discovery have been applied for over ten years now, as reviewed by Melville and collaborators (2009). Recently, Stokes et al. (2020) were the first to discover a new drug using the ML technique. Halicin, an inhibitor of c-Jun N-terminal kinase, was able to inhibit the growth of a broad spectrum of bacteria, both *in vitro* and *in vivo*. This drug was discovered from a screening campaign of over 6,000 molecules deposited in a drug repositioning bank, and has a structure that is considerably different from other antibiotics, acting on the dissipation of the potential of transmembrane pH in bacteria (Stokes et al., 2020).

As expected, no papers have been published on P2 receptors and drug discovery, but it is only a matter of time before new research emerges, as it is a promising area of research, and ML algorithms are already optimized and capable of learning. This is exemplified by Google Alphazero learning to play Go, which is considered to be one of the most complex games invented by humans (Silver et al., 2018; Rifaioglu et al., 2019).

Finally, ML algorithms have been applied in the identification of plant salinity stress (Feng et al., 2020), prediction of biological function based on structure (Liu et al., 2019), and regarding biological targets for natural molecules, like celastrol (Rodrigues et al., 2019). These studies revealed that ML can act as an important partner of MD. Rupp et al. (2014), for example, used ML to estimate the potential energy surfaces of natural molecules to speed up MD simulations (Rupp et al., 2014).

## Conclusions

Nature is a potential source for an almost infinite number of molecules. Natural products play an important role in drug discovery, even when we consider the obstacles presented by extracting, purifying, and separating active compounds. The high throughput screening that is usually applied by the pharmaceutical industry costs millions of dollars, and bioinformatics can test far more molecules in a faster and cheaper manner. In recent decades, this process has been used to search for and test natural products by *in silico* approaches. As a result, several naturally occurring molecules with action on P2 receptors have been discovered, which can be used as anti-inflammatory and antiplatelet agents. Moreover, several algorithms can also predict physicochemical, pharmacokinetic, and toxicity parameters. Therefore, it is expected that the introduction of artificial intelligence will lead to a more accurate selection of molecular hits and that, in the near future, machines will take the place of humans in the discovery of drugs concerning P2 receptors.

## Author Contributions

AA and LA formulated the manuscript. AA, RS, NF and LA wrote the manuscript.

## Funding

This work was supported by the Instituto Oswaldo Cruz (Fiocruz), Fundação de Amparo à Pesquisa do Estado do Rio de Janeiro (FAPERJ) and Conselho Nacional de Desenvolvimento Científico e Tecnológico (CNPq).

## Conflict of Interest

The authors declare that the research was conducted in the absence of any commercial or financial relationships that could be construed as a potential conflict of interest.
